# Left-Sided Poland Syndrome With No Hand Anomalies: A Case Report

**DOI:** 10.7759/cureus.33192

**Published:** 2022-12-31

**Authors:** Haider Sami, Husam Kivan, Sahar Al Hussein, Ammar Khawar, Ahmer Ashraf

**Affiliations:** 1 Internal Medicine, Lady Reading Hospital, Peshawar, PAK; 2 Medicine, OnDokuz Mayis University, Samsun, TUR; 3 General Medicine, Damascus University, Damascus, SYR; 4 Internal Medicine, Mayo Hospital, Lahore, PAK

**Keywords:** reconstructive breast surgery, reconstructive surgery, congenital, surgery, poland syndrome

## Abstract

A 22-year-old female presented to the surgical outpatient department with a complaint of left-breast hypoplasia. Upon physical examination, the left anterior chest wall was depressed, the left pectoral region was flattened, and the nipple was displaced. The absence of the pectoralis major sternocostal head was visible during shoulder abduction. Physical examination of the hands did not show any signs of ipsilateral digital abnormality. Chest X-ray revealed hyper translucent left-sided hemithorax with crowding of ribs and faint left breast soft tissue. A computed tomography scan (CT scan) reported a complete non-visualization of the left-sided pectoralis major, minor, and serratus anterior. Hence, a diagnosis of Poland syndrome involving left hemithorax in a female patient was established. The patient decided to have reconstructive surgery for purely cosmetic reasons.

## Introduction

Poland syndrome (PS) is an uncommon chest wall developmental abnormality that classically presents ipsilateral agenesis/hypoplasia of sternocostal head of pectoralis major, multiple rib abnormalities, elevated and rotated scapula (Sprengel deformity), hypoplasia of nipple or breast, absence of subcutaneous fat and ipsilateral digital abnormalities (brachydactyly, syndactyly) [1]. A constellation of all these symptoms is rarely found in a single individual [2,3]. Moreover, the condition is more frequent among men [4] and usually involves right hemithorax unilaterally [5]. Through this case report, we want to elaborate on the presentation of PS in a female and highlight the fact that PS can present without classical hand deformity.

## Case presentation

A 22-year-old woman presented to the surgical outdoor patient department complaining of left breast hypoplasia. She has had this problem since the time of puberty. On physical examination, an asymmetrical anterior chest wall with left-sided depression was observed. There was an upward displacement of the left nipple. The absence of the sternocostal head of pectoralis major was observed upon abduction of shoulders. Clinical defects, such as brachydactyly or syndactyly, were not noted upon hand examination. Moreover, she was born from a consanguineously married couple. There were no remarkable findings on examination of other systems, namely the nervous, gastrointestinal, respiratory, and urogenital systems.

**Figure 1:**
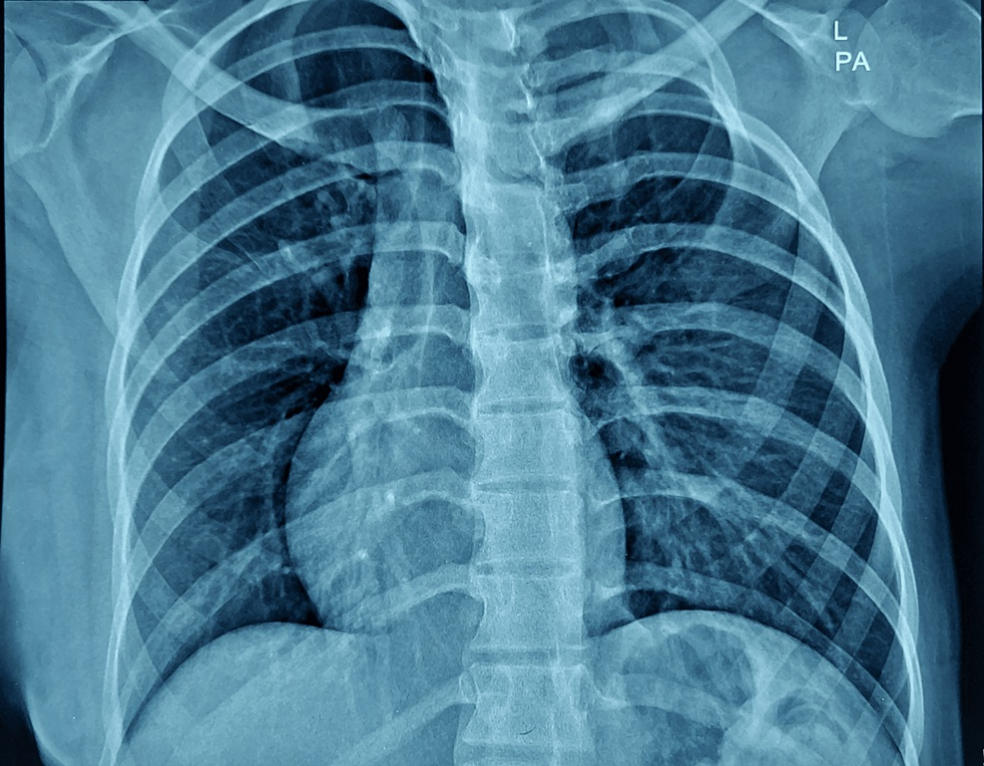
Chest X-ray PA view PA: Posteroanterior The left hemithorax is hyper-translucent, secondary to the absence of the left pectoralis muscle and anterior ends of four ribs (2nd to 5th). The heart and mediastinum are shifted to the right due to chest wall deformity.

Chest X-ray showed hyper translucent left-sided hemithorax with crowding of ribs and faint left breast soft tissue. It further explained that the apex of the heart was left-sided. These findings were confirmed on ultrasound. The heart was normal in size and shifted toward the right side. No gross active lung pathology was noted. Costophrenic angles are clear. Hila have no significant finding (Figure 1)

**Figure 2:**
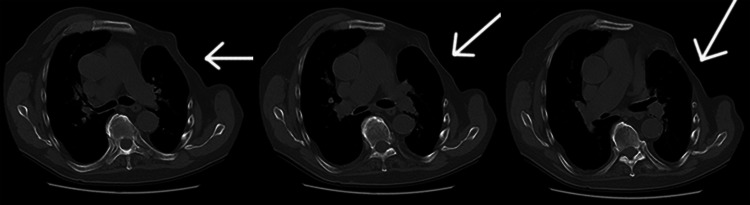
Non-contrast CT chest (axial view) The arrows show a complete absence of the left pectoralis major muscle and the rudimentary pectoralis minor muscle.

CT chest revealed complete non-visualization of the left-sided pectoralis major, minor, and serratus anterior muscle. Rudimentary left breast tissue was visualized (Figure 2). The right breast was normal. Right-sided pectoralis major, minor, and serratus anterior muscle were normal. Chest X-ray correlation showed hypoplastic 1st four ribs on the left side. The rest of visualized lung fields were normal.

A diagnosis of left-sided Poland syndrome was established, which was not associated with the classical hand deformity. The patient was concerned about the physical aspect of the anomaly, so different reconstructive methods were explained, including reconstruction by implants or fat grafting. Finally, reconstruction by Becker’s implant was decided.

## Discussion

Poland's syndrome (also Poland syndrome, Poland sequence, Poland's anomaly, and Poland's syndactyly) is a rare congenital syndrome that was first described in 1841 by Sir Alfred Poland. It predominantly affects males with an estimated incidence of 1/30000 to 1/32000 live births [5,6]. Sporadic cases are in majority. Familial cases have also been documented. This syndrome tends to involve the right side of the body and is usually unilateral [7,8]. Involvement of the left side of the body has only been reported in approximately 25% of the cases [8]. Hand involvement is prevalent.

Poland syndrome is generally a defect of sporadic origin. Cases pointing toward genetic inheritance are rare. It has been hypothesized that the damage develops during the sixth week of pregnancy as a result of a regional vascular defect in the subclavian artery [9]. It is the very period where two heads of the pectoralis major muscle split and the development of tissue between digits take place. However, the definite cause explaining the detailed pathogenesis behind Poland syndrome has yet to be discovered.

Poland syndrome generally involves the right side of the body and gender predisposition is in favor of the male sex. Studies conducted by different institutes revealed that males are affected three times as often as females with right-sided deformities twice as common as left-sided deformities [4]. However, this data is confounded by the fact that most of the male patients remain undiagnosed and are only documented when there is an associated functional limitation.

Numerous characteristics of Poland syndrome can be identified, including visceral defects like hernia, renal agenesis, and atrial septal defect. According to reports, Poland syndrome has also been linked to blood dyscrasias. There have also been reports of acquired perforating dermatosis, congenital hemangiomas, psoriasis vulgaris, cutaneous diffuse neurofibroma, and café-au-lait spots [10]. These features carry the potential to redefine Poland's syndrome itself. Moreover, an extensive investigation should be carried out on such patients to further elaborate on the association of different features [11].

Other recognized diseases that can be included in the differential diagnosis for Poland's syndrome include Becker's nevus syndrome, cutaneous hamartoma/nevus, webbed neck with restricted movement, Mobius syndrome, Becker's Sprengel deformity, small and high scapula, Klippel-Feil syndrome, scoliosis, paralysis of multiple cranial nerves, anterior thoracic hypoplasia, and Amazon syndrome. Additionally, traumatic chest injury cases should also be explored extensively because Poland syndrome can mimic traumatic chest injury as well [12].

Treatment of Poland syndrome depends upon various factors such as age, sex, type of deformity, and associated functional limitation [13]. Surgery for the thoracic anomaly and surgery for the hand anomaly are the two main surgical procedures used to address Poland anomalies. The thoracic anomaly often requires just cosmetic surgery. Paroxysmal motions of the chest wall and increasing lung herniation are two additional reasons for surgery. The functional and anatomical defects of the chest can be corrected using a variety of reconstructive techniques, including lipofilling, tailored silicone prostheses, and flaps (latissimus dorsi, rectus abdominis, and omental). Prior to any reconstructive treatment, it is essential to evaluate the latissimus dorsi's health because it is the most frequently employed flap due to its location. Surgical reconstruction for adults is often performed in the first stage and the second stage for children [14].

The other dimension while considering surgical intervention is hand anomaly. Depending on the particular nature of the deformity, surgical intervention for hand anomalies should focus on reconstructing a functional hand. In a wide spectrum of symbrachydactyly, the goal would be to gain pinch and grasp maneuvers, Digital separation is recommended in all cases. Ideally, repair of syndactyly should be done at preschool age [15].

## Conclusions

Poland syndrome is an uncommon congenital musculoskeletal anomaly with a significant spectrum of presentations. It is classically characterized by aplasia or hypoplasia of the chest wall muscle and is variably associated with ipsilateral limb anomalies. Presentation of hand defects can be variable, ranging from syndactyly to phocomelia. However, classical defining features of Poland syndrome, like limb abnormality, may not be present in all cases. Moreover, the male gender is more predisposed to Poland syndrome. Mostly, Poland syndrome is treated surgically with a strong emphasis on aesthetics.
